# Heart Disease and Pectus Excavatum: An Underestimated Issue—Single Center Experience and Literature Review

**DOI:** 10.3390/life14121643

**Published:** 2024-12-11

**Authors:** Alice Ravasin, Domenico Viggiano, Simone Tombelli, Luca Checchi, Pierluigi Stefàno, Luca Voltolini, Alessandro Gonfiotti

**Affiliations:** 1 Thoracic Surgery Unit, Careggi University Hospital, 50134 Florence, Italy; viggianod@aou-careggi.toscana.it (D.V.); simone.tombelli@unifi.it (S.T.); luca.voltolini@unifi.it (L.V.); a.gonfiotti@unifi.it (A.G.); 2 Arrhythmia Unit, Careggi University Hospital, 50134 Florence, Italy; 3 Cardiac Surgery Unit, Careggi University Hospital, 50134 Florence, Italy; 4 Department of Experimental and Clinical Medicine, University of Florence, 50134 Florence, Italy

**Keywords:** pectus excavatum, heart disease, pectus excavatum repair, arrhythmias, mitral valve prolapse, pericarditis

## Abstract

Pectus excavatum (PE) can be associated with either congenital or acquired heart disease. This study highlights the importance of PE surgical repair in cases of severe chest depression on the heart in underlying cardiac diseases exacerbating cardiopulmonary impairment. From January 2023 to March 2024, four male patients underwent PE repair, having heart disease including pericarditis, mitral valve prolapse, ventricular fibrillation arrest and type 1 second-degree atrioventricular block. PE severity was determined by the Haller index (HI). Preoperative assessment included a pulmonary function test, chest computed tomography and cardiac evaluation. The Nuss procedure was performed in three patients, whereas, in one patient, it was performed in combination with a modified Ravitch procedure. The median HI was five. The median time of chest tube removal was 6.5 days. Postoperative complications were prolonged air leak, atrial fibrillation and atelectasis. The median length of hospital stay was 19.5 days, and no 30-day postoperative mortality was recorded. In all patients, surgical repair helped to resolve the underlying cardiological issues, and surgical follow-ups were deemed regular. PE is generally an isolated congenital chest wall abnormality, and, when associated with a heart disease, it can have severe life-threatening hemodynamic consequences due to mechanical compression on the heart for which surgical corrections should be considered.

## 1. Introduction

Pectus excavatum (PE) is the most common chest wall congenital deformity. The incidence is around 1 in 400 children, and it occurs four times more frequently in males than in females [1]. It appears at a young age and can increase progressively with growth. A familial tendency has been observed, although no specific genetic defect has been identified yet. Generally, it is an isolated congenital abnormality, but, rarely, concurrent congenital heart disease may be found in these patients [2].

Symptoms may initially be mild and present only under strain, but they may worsen as the chest deformity increases. In milder cases, the defect may only cause aesthetic issues, which affects the quality of life of young patients but do not cause any pathophysiological alteration. The anterior chest wall depression, especially when severe, leads to a reduced distance between the sternum and column vertebrae, which in turn is associated with displacement of the heart to the left hemithorax and rotation with compression of heart chambers, causing rhythm changes [3] that may result in significant cardiorespiratory impairment [4]. PE is common in patients with connective tissue disorders; in particular it has been observed in two-thirds of patients with Marfan syndrome (MS), an autosomal dominant disease that particularly affects the cardiovascular system (e.g., aorta dilation and mitral regurgitation), the skeletal system (e.g., chest wall deformities), the respiratory and ocular systems [5]. The aim of this study is to describe our experience of concurrent PE and cardiac disease, emphasizing the importance of surgical correction in symptomatic patients with a severe chest wall depression, causing cardiopulmonary complications secondary to the mechanical compression of the heart.

## 2. Materials and Methods

We reviewed all patients undergoing PE repair at Careggi University Hospital of Florence, Italy, and we selected those with concurrent heart diseases.

**Figure 1:**
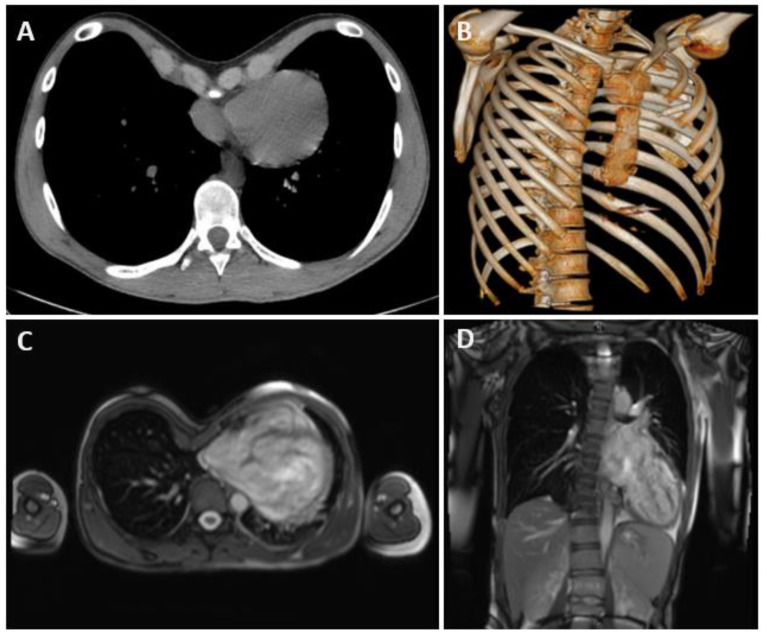
(**A**,**B**) Chest computed tomography and 3D reconstruction showing severe pectus excavatum (PE) in patient with Micra leadless pacemaker (MLP). (**C**,**D**) Cardiac magnetic resonance in a Marfan patient with severe PE and heart displaced to the left.

Preoperative assessment included a pulmonary function test, chest computed tomography, cardiac evaluation with electrocardiography, echocardiography, a cardiopulmonary exercise test and cardiac magnetic resonance (Figure 1).

A literature review was performed on the PubMed database using keywords related to pectus excavatum, pectus excavatum repair and heart disease.

### Surgical Procedure

All patients were placed in a supine position, with arms adducted alongside the body and blankets utilized as necessary to appropriately elevate the chest. The sternum was lifted with a steel wire using the crane technique [6]. The Nuss procedure was performed through two lateral skin incisions of approximately 4–5 cm, on each side of the inferior edge of pectoralis major muscle, creating two retromuscular pockets. Thoracoscopy was carried out by inserting a 5 mm trocar on the right-middle axillary line, and carbon dioxide (CO_2_) insufflation was used to find and safely dissect the retrosternal space. An introducer was inserted into the chest through the right surgical access, and the dissection was conducted under thoracoscopic vision by means of rotational and forward movements of the dissector (with the tip facing forward) through the mediastinum just above the pericardium. Once reaching the left selected intercostal space, the tip of the introducer was brought out through the left surgical incision. After the substernal tunnel was created, one side of the suction connecting tube was linked to the introducer tip, and the other side was connected to the right end of the curved bar [7]. The introducer was carefully pulled backward from left to right followed by the tube, creating a path for the bar to pass with the concave side up. Then, the bar was rotated 180 degrees using two tools (flippers), which allowed the synchronized rotation of the bar, resulting in its final convex position correcting the sternal deformity, pushing it forward. Bar stabilization is essential for a successful outcome to avoid rotation. At the end of the procedure, a chest tube was left in place.

## 3. Results

Between January 2023 and March 2024, four patients with PE and concurrent heart disease underwent surgical repair at Careggi University Hospital of Florence, Italy. All patients were male, and the median age was 18.5 (range 15–52) years. Patients’ clinical data are reported in Table 1.

All patients underwent the Nuss procedure for the PE correction. In one case, the Nuss technique was not effective due to the patient’s age and the rigidity/stiffness of the chest wall. Therefore, in this case, the two previously implanted bars were removed and a modified Ravitch technique was performed through a median longitudinal skin incision of approximately 10 cm; once reaching the costal plane, the aberrant cartilages of the sixth, seventh and eighth costal arches were removed bilaterally, a sternal wedge was created, and a Nuss bar was finally inserted to ensure stabilization.

One case involved a 15-year-old boy with MS who underwent the simultaneous minimally invasive repair of pectus excavatum (MIRPE) and mitral valve repair plus annuloplasty. In this case, a single bar was first positioned to obtain an initial correction of the sternal defect, resulting in a medial shift of the mediastinum to allow mitral valve repair. The right minithoracotomy at the fourth intercostal space was then enlarged, and the pericardium was opened. After systemic heparinization, peripheral cannulation was performed, and cardiopulmonary bypass started. After aortic cross-clamp and antegrade cold blood cardioplegia, the mitral valve was exposed through left atriotomy. Mitral plasty was performed through triangular resection and the folding of P2 (middle scallop) and the implantation of a pair of Gore-Tex 4-0 Neochord for the posterior flap in P2. Annuloplasty was performed with Corcym Memo 4D ring n.38 with detached U-shaped stitches in Ti-Cron 2-0. Once the cardiac correction was completed, two further bars were positioned with the Nuss technique for the optimal correction of the PE. The minithoracotomy was covered with a Permacol biological patch, fixing it perimetrically with transcostal vicryl threads.

No intraoperative complications occurred in the present case series. The median operating time was 92.5 (range 55–455) minutes. The median time of chest tube removal was 6.5 (range 3–29) days, and the median length of hospital stay was 19.5 (range 4–36) days, respectively.

In the patient with concurrent cardiac surgery, the postoperative course was characterized by a prolonged postoperative air leak, requiring surgical revision and video-assisted thoracic surgery (VATS) bullectomy and pleurodesis. Another patient, a 16-year-old boy, underwent subcutaneous implantable cardioverter-defibrillator (S-ICD) 11 days after MIRPE [8].

No 30-day postoperative mortality was recorded in the case series. In all patients, surgical repair helped to resolve the underlying cardiological issues, and surgical follow-up was deemed regular. In the 52-year-old patient undergoing the combined Ravitch and Nuss procedure, no further episodes of pericarditis were reported after PE repair.

**Figure 2:**
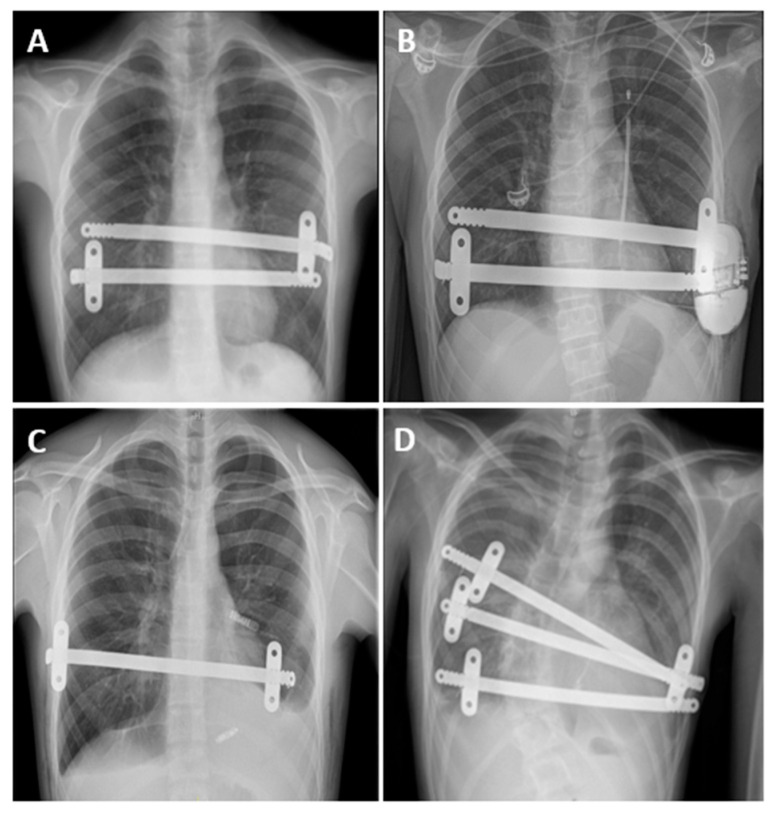
(**A**) Chest radiograph showing pectus repair with the insertion of two retrosternal metal bars and (**B**) after subcutaneous implantable cardioverter-defibrillator (S-ICD) implantation. (**C**) Chest radiograph showing pectus repair with the insertion of one retrosternal metal bar and MLP. (**D**) Chest radiograph showing pectus repair with the insertion of three retrosternal metal bars in a Marfan patient with a residual right-sided postoperative pneumothorax.

Table 2 summarizes operative and postoperative data. Figure 2 shows some of the patients’ postoperative chest X-rays.

## 4. Discussion

PE is the most common chest wall deformity and can be associated with congenital or acquired heart disease that may also require surgical correction [2,9]. In the present case series, we described our clinical experience of patients with concomitant heart disease undergoing PE repair. No postoperative mortality was reported, the postoperative complication rate was low, and, in all patients, surgical PE repair helped to resolve the underlying cardiological issues. Although the sample size was small, our results are in line with the current literature data.

Sacco Casamassima et al. [10] reported a case series of nine patients with connective tissue disorders and severe PE, who underwent a modified Nuss procedure combined with open heart surgery (i.e., aortic root replacement, mitral valvuloplasty). The authors found that the simultaneous repair of PE and open-heart surgery was safe and effective, although they recommended reserving the decision to perform a single-stage procedure until after the cardiac procedure has been completed. Similarly, in a study conducted by Dimitrakakis et al. [11], the authors reported the successful use of the Nuss procedure in combination with mitral valve surgery to treat patients with both severe PE and mitral valve disease. In this study, the authors emphasized the benefits of this dual approach, which allows for the correction of both the cardiac and chest wall abnormalities in a single operation, minimizing the need for multiple surgeries and reducing the associated risks and recovery times. Furthermore, a 2017 review conducted by Raffa et al. [12] examined the outcomes of aortic surgery in Marfan syndrome patients who also had severe PE. Highlighting the challenges of managing these patients in a coordinated approach, Raffa et al. highlighted that successful aortic surgery requires careful planning and tailored strategies to avoid complications and optimize outcomes.

In the present series, the case of a 15-year-old patient with MS underwent a single-stage PE and mitral valve repair. We planned a combined procedure because the abnormal chest deviation determined significant displacement of the heart and great vessels towards the left hemithorax, thus hindering optimal cardiac exposure and precluding a two-stage surgery.

All things considered, the incidence of cardiovascular diseases that require open-heart surgery and concomitant PE repair continues to be matter of debate due to its rarity and uncommon occurrence. No consensus has yet been reached in the literature regarding the ideal age of repair, whether the repair should be performed simultaneously or in a staged approach, and which is the optimal surgical technique (i.e., Ravitch versus Nuss) [13,14]. In most cases, median sternotomy is the preferred open-heart surgery approach because it provides optimal exposure and access to the entire heart. However, in severe cases of PE, sternal rotation and heart dislocation towards the left hemithorax may not allow a median sternotomy to achieve optimal cardiac exposure and alternative surgical approaches may be necessary [9,10,11,15,16,17]. In our case, we performed mitral valve repair through right minithoracotomy, and, to obtain an adequate cardiac exposure, a Nuss bar was inserted previously.

Cardiac compression can lead to postoperative hemodynamic instability if PE is left uncorrected [18,19]. Stephens at al. presented a case of a young female with MS and associated severe PE who underwent the elective replacement of an aortic root aneurysm. Immediately after sternum closure, the patient became hypotensive and was unresponsive to inotropic support and preload manipulation, thus requiring simultaneous unplanned PE repair [20].

Furthermore, the concurrent presence of congenital or acquired cardiac disease in severe PE compressing the heart must not be underestimated. In PE, the depression of the anterior chest wall is caused by dorsal deviation of the sternum and the third to seventh ribs or costal cartilages, while the manubrium, first and second costal cartilages are generally not involved [21]. PE repair should be avoided in infants and children and may be delayed until the end of the second growth spurt due to the risk of recurrence and the changes that occur during puberty.

Adolescents are considered ideal candidates for a MIRPE approach [22,23], while the stiffness of an adult chest wall and its limited theoretical remodeling historically favored the use of the more invasive traditional Ravitch procedure [24,25]. In the present case series, the 52-year-old man underwent a modified Ravitch procedure due to the relevant rigidity of the anterior chest wall. Preoperatively, the patient reported a progressive worsening in respiratory mechanics that was linked to the thoracic anomaly, as the bone structures were determining a compression of the pericardium. Although there is no clear evidence in the literature regarding constrictive pericarditis and PE, Wolfenden et al. described a case of constrictive pericarditis in PE, assuming that the constriction was secondary to an organized traumatic pericardial hematoma immediately behind the pectus deformity [26]. Similarly, Gerfer et al. described a rare case of constrictive pericarditis with a huge pericardial cyst and PE compressing the heart [27]. In any case, the depression of the sternum leads to a reduction in thoracic volume and may compress the heart with its possible displacement towards the left hemithorax [28].

PE severity is determined by the Haller index (HI), and surgical correction is generally considered for HI ≥ 3.25 [29]. In our series, the median HI was 5.0 (range 4.0–6.8), and all patients presented cardiovascular issues warranting surgical repair as recommended by the evaluation criteria reported by Stephens et al. [30].

Symptoms due to PE deformity are rarely observed during early childhood, but they may worsen during adolescence and adulthood as a result of less flexibility of the chest wall and a decrease in compensatory mechanisms [31]. The most common symptoms include easy fatigue, reduced exercise ability, dyspnea, chest pain and palpitations [32]. In severe cases, the mechanical compression directly exercised by the depressed sternum on the heart may cause cardiac arrhythmias and severe, potentially life-threatening hemodynamic consequences, reducing stroke volume and cardiac output [33]. Dysrhythmias may be seen on the electrocardiogram (ECG) as a first- or second-degree heart block and bundle branch block, ventricular tachycardia, atrial fibrillation, incomplete left bundle branch block, or Wolff–Parkinson–White syndrome [34]. In our series, we reported the case of a 21-year-old patient with severe PE and a history of second-degree atrioventricular block type 1.

In the 2013 study conducted by Tran et al. [35], the authors investigated the association between lone atrial fibrillation (AF) and PE. The study found that patients with PE were more likely to develop lone AF even in the absence of other cardiac conditions, thus suggesting that the structural abnormalities of the chest wall in PE may contribute to altered cardiac mechanics or autonomic function, potentially increasing the risk of atrial arrhythmias. Furthermore, Ferraz at al. [36] documented the case of a young woman with severe PE, which led to leftward displacement of the heart, with compression and deformation of the right cavities causing frequent symptomatic ventricular extrasystoles. Hence, the authors suggested that surgical PE repair should be considered in cases of structural and hemodynamic changes.

Still, the literature data are poor concerning the management of arrhythmia or cardiac arrest in PE patients, and little is known about the best treatment in these cases. In our case series, a previously healthy 16-year-old boy presented with ventricular fibrillation and cardiac arrest. We postulated that the arrhythmic event may have been caused by PE compression of the right ventricle. This is in accordance with other authors who have reported that surgical PE repair may solve the compression exerted on the right ventricle by the depressed chest wall, preventing the induction of cardiac arrhythmias [37,38,39]. However, lacking certain data regarding PE repair as a reversible cause of sudden cardiac arrest, according to the current European Society of Cardiology (ESC) guidelines [40], a S-ICD was implanted in our patient as a secondary prevention. In addition, the patient received genetic counseling. Subsequent testing identified the presence of a pathogenic variant in desmoglein-2-gene (DSG-2), which is a gene associated with non-dilated left ventricular cardiomyopathy (NDLVC).

### Limitations

This work presented a single-center retrospective case study with a very small group of patients—two adolescents and two adults. While the rarity of the co-existing conditions naturally restricts the sample size, it also highlights the complexity and heterogeneity of the cardiac conditions we encountered. The different surgical techniques performed (Nuss versus Ravitch) add complexity to the evaluation and the interpretation of outcomes. A bias of this study is the unavailability of one-year follow-up data for one of the four patients, as, in this case, one year had not passed yet since surgery.

## 5. Conclusions

PE is generally an isolated congenital chest wall abnormality, and its repair is usually performed for aesthetic reasons and psychosocial impact. However, the mechanical compression exerted by severe PE on the heart must not be underestimated in concomitant congenital or acquired cardiac disease. Symptomatic patients with an underlying heart disease exacerbated by a depressed chest wall may have severe and life-threatening hemodynamic consequences for which surgical corrections should be considered.

## Figures and Tables

**Table 1:** Patients’ clinical data.

Case	Age (yr)	HI	Heart Disease	Implantable Medical Devices	ASA	Comorbidities
1	16	5	VF arrest	S-ICD	3	-
2	15	6.8	MVP	-	3	MS, Scoliosis, PA
3	21	5	II° AVB	MLP	3	ADHD, BD, Asthma
4	52	4	Pericarditis	-	3	Mondor Syndrome

HI: Haller index; ASA: American Society of Anesthesiologists; VF: ventricular fibrillation; S-ICD: subcutaneous implantable cardioverter-defibrillator; MVP: mitral valve prolapse; MS: Marfan syndrome; PA: pilocytic astrocytoma; II° AVB: second-degree atrioventricular block; MLP: Micra leadless pacemaker; ADHD: attention-deficit hyperactivity disorder; and BD: Bipolar disorder.

**Table 2:** Operative and postoperative data.

Case	Pectus Technique	Operation Time (min)	Number of Bars Placed	Resection of Costal Cartilage	Wedge Resection of the Sternal Body	Postoperative Complications	Chest Tube Duration (Days)	LOS (Days)
1	Nuss	80	2	-	-	-	3	28
2	Nuss	455	3	-	-	PAL, fever	29	36
3	Nuss	55	1	-	-	Transitory fever	4	4
4	Modified Ravitch + Nuss	105	1	6th, 7th, and 8th ribs bilaterally	Yes	AF, Atelectasis	9	11

LOS: length of hospital stay; PAL: prolonged air leak; and AF: atrial fibrillation.

## Data Availability

All relevant data are within the manuscript.
